# In silico study to identify novel potential thiadiazole-based molecules as anti-Covid-19 candidates by hierarchical virtual screening and molecular dynamics simulations

**DOI:** 10.1007/s11224-022-01985-1

**Published:** 2022-06-15

**Authors:** Huda R. M. Rashdan, Aboubakr H. Abdelmonsef

**Affiliations:** 1 Chemistry of Natural and Microbial Products Department, Pharmaceutical and Drug Industries Research Institute, National Research Centre, Dokki, 12622 Cairo Egypt; 2 Chemistry Department, Faculty of Science, South Valley University, Qena, 83523 Egypt

**Keywords:** 1,3,4-Thiadiazoles, Covid-19 pandemic, Molecular dynamics, SARS-CoV-2, Hydrazonoyl halides, Molecular docking

## Abstract

In the present study, a new category of 1,3,4-thiadiazoles was developed by submitting methyl 2-(4-hydroxy-3-methoxybenzylidene) hydrazine-1-carbodithioate to react with the appropriate hydrazonoyl halides in presence of few drops of diisopropyl ethyl amine. The chemical structures of the newly synthesized derivatives were inferred by means of their micro-analytical and spectral data. Utilizing combined molecular docking and molecular dynamics techniques, the binding affinities and features of the synthesized compounds were evaluated against four SARS-CoV-2 target enzymes, namely, main protease (M^pro^), papain-like protease (PL^pro^), RNA-dependent RNA polymerase (RdRp), and receptor-binding domain (RBD) of the spike protein. Compound **7** demonstrated promising binding affinities with the target enzymes M^pro^, PL^pro^, RdRp, and RBD with docking scores of −11.4, −9.4, −8.2, and −6.8 kcal/mol, respectively. In addition, compound **7** exhibited MM-GBSA//100 ns MD docking score of −35.9 kcal/mol against M^pro^. Structural and energetic analyses revealed the stability of the **7**-M^pro^ complex over 100 ns MD simulations. In addition, compound **7** obeyed Lipinski’s rule of five, as it has acceptable absorption, distribution, and oral bioavailability inside the body. Therefore, compound **7** is considered as a promising starting point for designing potential therapeutic agents against Covid-19.

**Supplementary Information:**

The online version contains supplementary material available at 10.1007/s11224-022-01985-1.

## Introduction

A new strain for SARS-CoV-1 identified recently as SARS-CoV-2 in late December 2019 resulted in serious physical and psychological damages to the human health; a massive outbreak initially in Wuhan, China, and spread rapidly in different nations around the global in a short time [1, 2]. The World Health Organization (WHO) declared this highly infectious respiratory disease Covid-19 as a pandemic [3]. It is acceptable to think that a sufficient understanding of SARS-CoV-2 and the full clinical picture of the resulting Covid-19 disease will take some time. However, the first detected clinical sign of Covid-19 was pneumonia [4]. Recently, asymptomic infections and gastrrointestinal symptoms were also reported especially among young children [5, 6]. Pneumonia mostly appeared in the second or third week of the infection. Decreased oxygen saturation, blood gas deviations, and changes visible through chest X-rays are prominent signs of viral pneumonia. In addition, lymphopenia documented to be common, and inflammatory markers (Proinflammatory cytokines and CRP) are elevated. Consequently, investigation of anti-Covid-19 therapeutic agents became an urgent demand and attracted more interest recently owing to the lack of specific drugs for the treatment of Covid-19 [7, 8]. Nevertheless, several existing drugs are available only to overcome the clinical symptoms of Covid-19.

On the other hand, 1,3,4-thiadiazole moieties have been reported for their pharmaceutical properties. Many antiviral drugs like acetazolamide, besaglybuzole (glybuzole), and furidiazine (triafur) were reported to append the 1,3,4-thiadiazole in their constructors[9–13].

Urgent needs for develoment novel anti-Covid19 agents have directed us to synthesize some new bioactive heterocyclic molecules. In the present study, we aimed to identify potential Covid-19 inhibitors through a computer-based molecular docking and molecular dynamics techniques [14–16]. In addition, ADMET (absorption, distribution, metabolic, excretion, and toxicity) and pharmacokinetics parameters of the prepared ligand molecules were performed to identify their drug-likeness properties [17].

## Results & discussion

### Chemistry

**Scheme 1:**
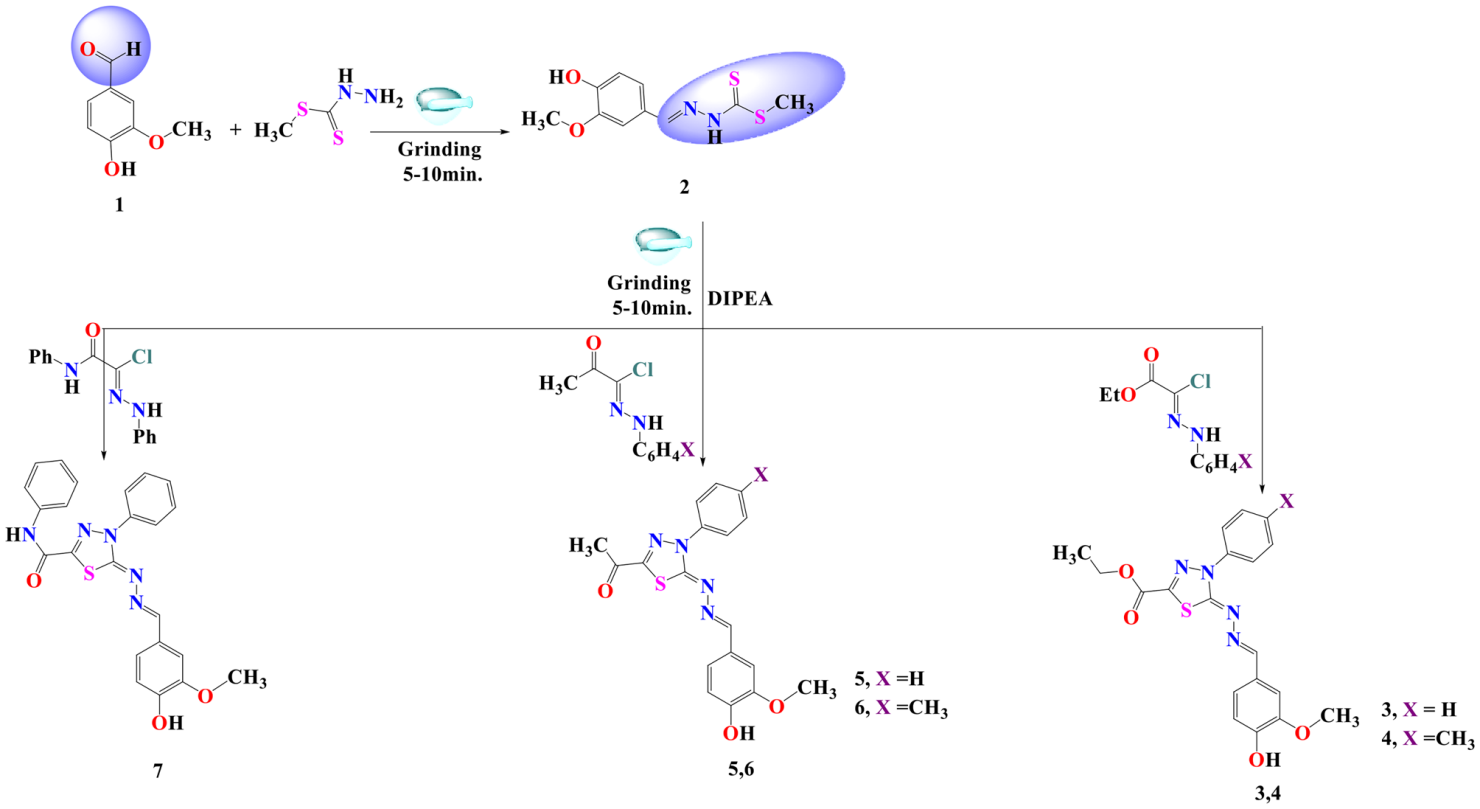
Synthetic procedures of the desired 1,3,4-thiadiazoles **3–7**

Methyl 2-(4-hydroxy-3-methoxybenzylidene)hydrazine-1-carbodi-thioate (**2**) acts as key molecule for the design of new desired 1,3,4-thiadiazole compounds. It is allowed to react with a selected group of hydrazonoyl halide derivatives by grinding method “grindstone chemistry” under solvent-free conditions with the addition of catalytic amount of DIPEA (diisopropyl ethyl amine) to afford the target molecules **3–7**, (Scheme 1).

**Fig. 1:**
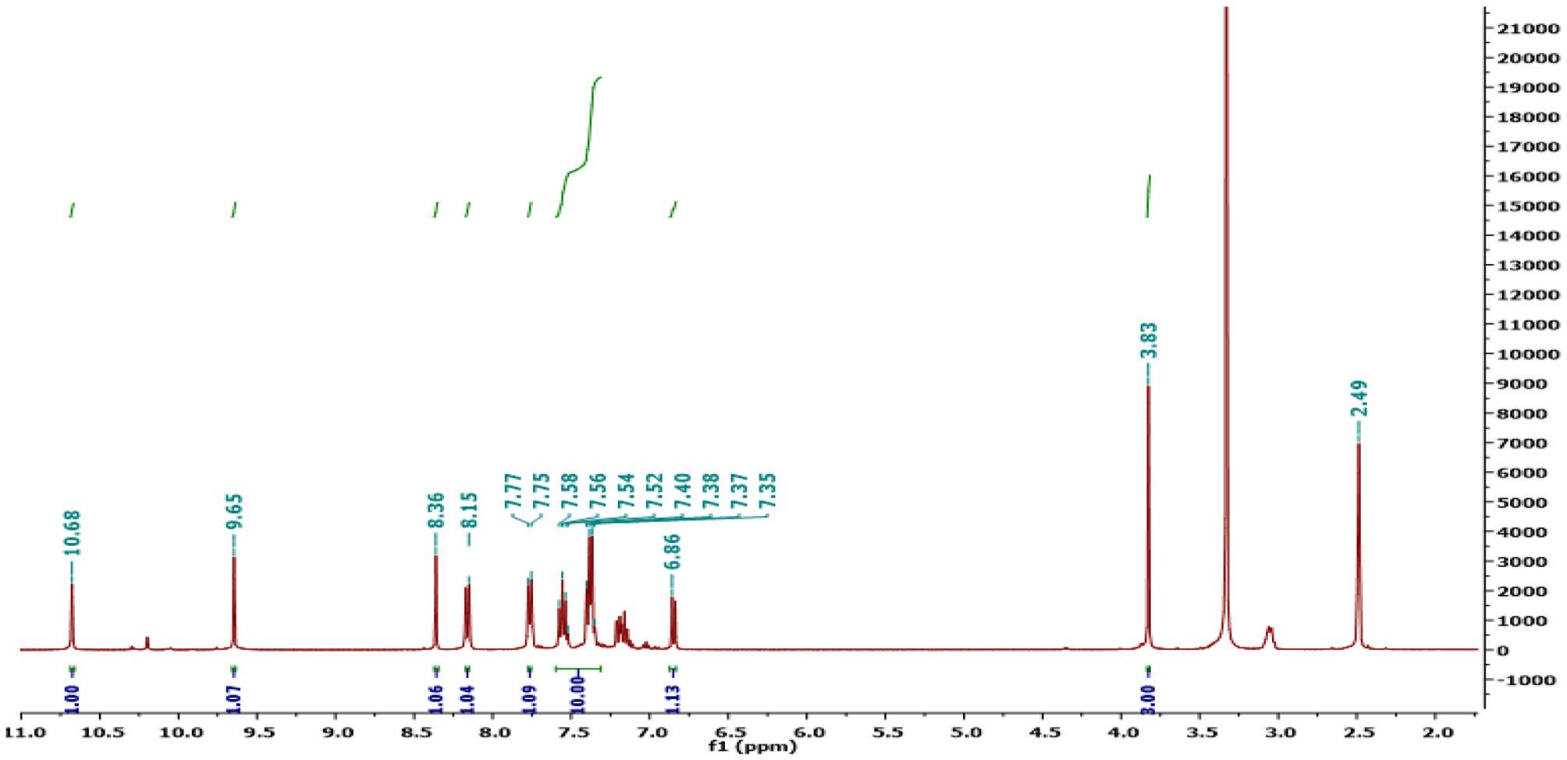
^1^H.^−^NMR spectrum of the target molecule **7**

The chemical structures of all newly prepared molecules are affirmed by spectral and elemental data. For instance, IR spectrum of the target molecule **7** revealed a strong broad absorption band at *v* 3337 assigned for NH group. Additionally, it showed a strong sharp absorption band at *v* 1681 attributed to the carbonyl group. Meanwhile, ^1^H-NMR spectrum exhibited singlet signal at δ 3.83 ppm represented the methoxy group along with multiplet signal at δ 6.86–7.85 ppm for aromatic protons. Also, it revealed doublet signal at δ 7.75 ppm attributed to the aromatic hydrogen and doublet signal at δ 8.15 ppm represented the aromatic hydrogen. Moreover, it showed three singlet signals at δ 8.36, 9.65, and 10.68 ppm for CH = N, OH and NH, respectively as illustrated in Fig. 1.

**Fig. 2:**
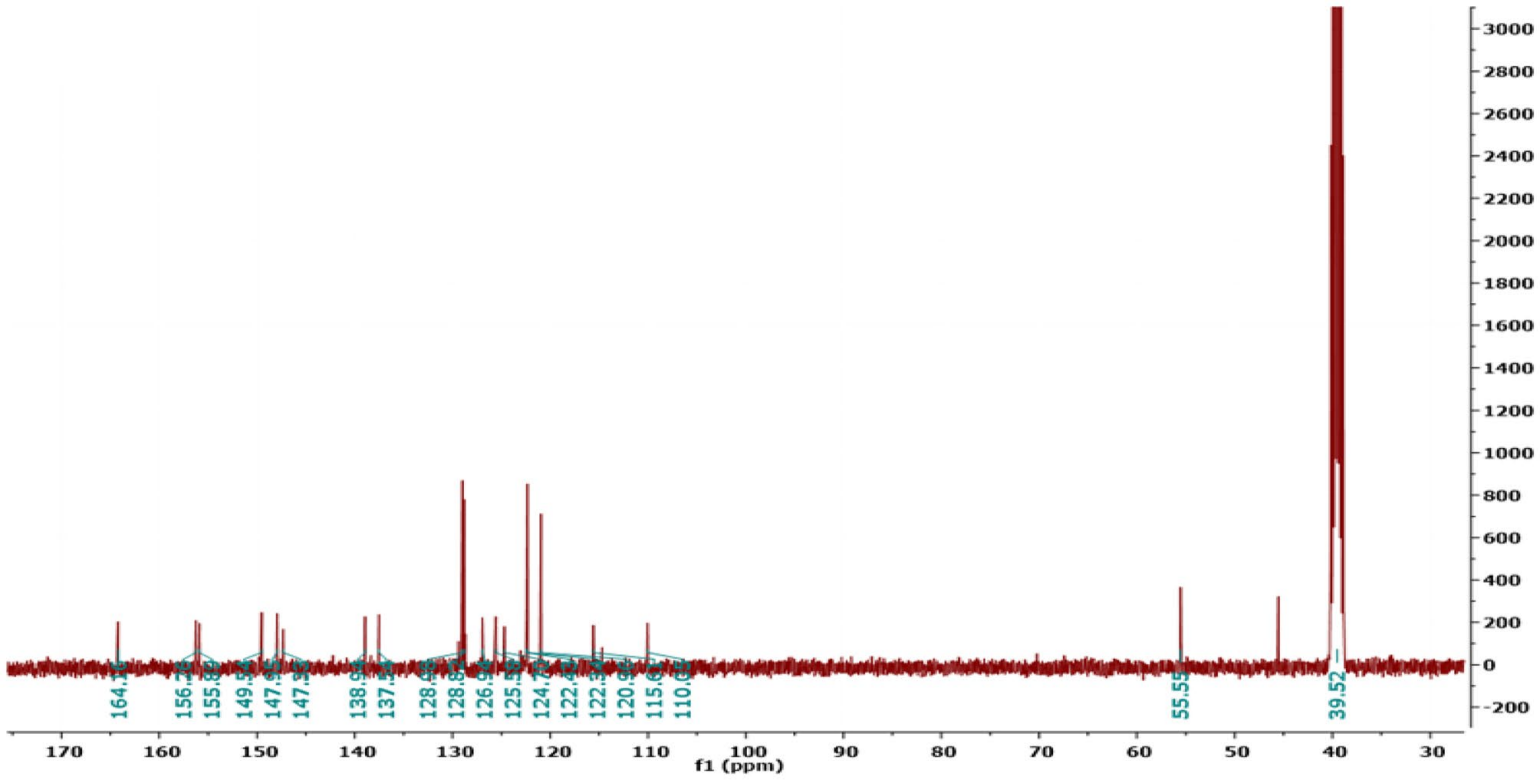
^13^C-NMR spectrum of target molecule **7**

Figure 2 revealed the significant signals of the target molecule **7** which confirmed the formation of the compound.

**Fig. 3:**
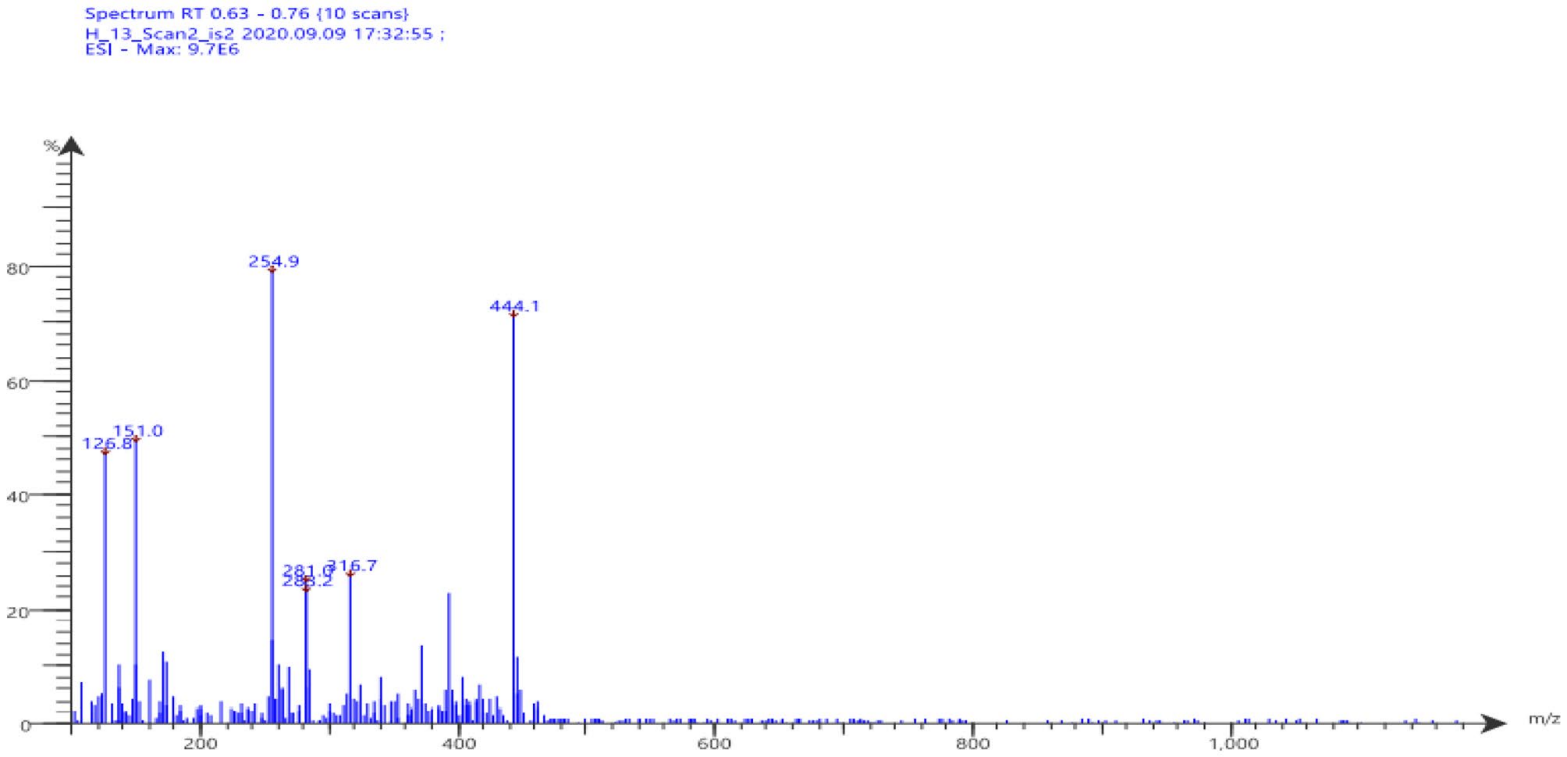
Mass spectrum of compound **7**

The chemical composition of the target molecule **7** was affirmed also by the mass spectrum (m/z 445) [M^+^], which agrees with its molecular formula C_23_H_19_N_5_O_3_S, as represented in Fig. 3.

### Molecular docking calculations

**Fig. 4:**
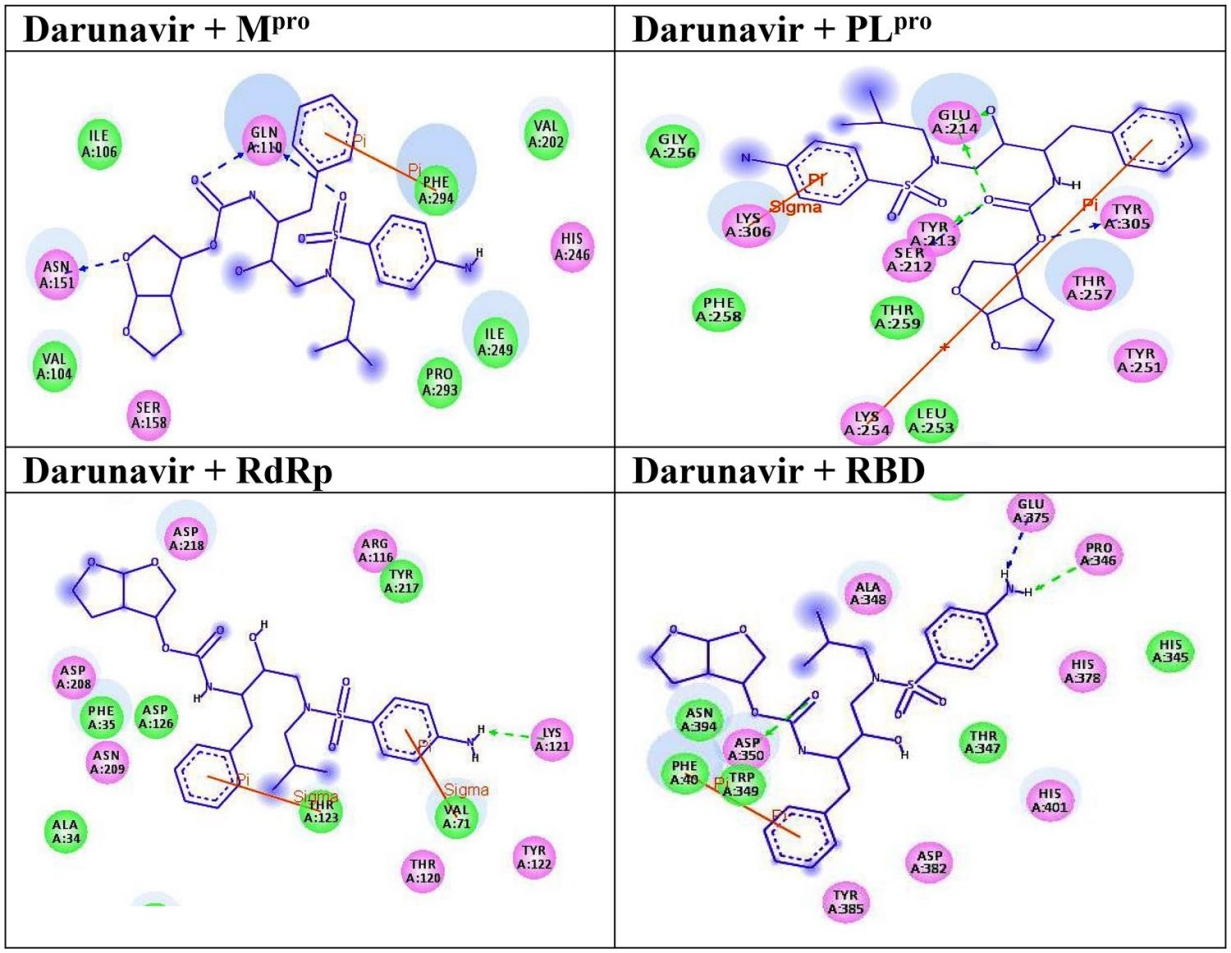
2D representations of interactions between the reference drug and the targets. Blue stick models represented the docked compounds, and colored balls represented the active site region. H-bond interactions are shown in green and blue dotted lines. π-interactions are shown in orange lines

The molecular docking technique [11, 18] was used to predict the binding modes and affinities of the newly synthesized compounds with SARS-CoV-2 targets M^pro^, PL^pro^, RdRp, and RBD of S-protein. The predicted docking scores are tabulated in Table 1. 2D (two dimensional) and 3D (three dimensional) representations of binding modes of best docked compound **7** inside the active site of M^pro^, PL^pro^, RdRp, and RBD are displayed in Fig. 4. The representations of the rest docked compounds against the targets are shown as Figs. S1–S4, respectively, in the Supplementary file section.

**Table 1:** Calculated binding energies (in kcal/mol) of synthesized compounds with the targets

**Compound**	**Docking Score (kcal/mol)**			
	**M**^**pro**^	**PL**^**pro**^	**RdRp**	**RDB**
**Darunavir**	−7.5	−7.2	−7.7	−6.6
2	−6.4	−5.6	−5.2	−5.7
3	−10.4	−8.0	−7.6	−5.4
4	−9.8	−7.6	−7.5	−6.5
5	−10.4	−8.0	−6.5	−5.3
6	−10.0	−6.6	−6.0	−5.4
7	−11.4	−9.4	−8.2	−6.8

It is observed from the data in Table 1 that compound **7** exhibited binding affinities against all the selected targets better than the reference drug (Darunavir) (Fig. 4).

**Fig. 5:**
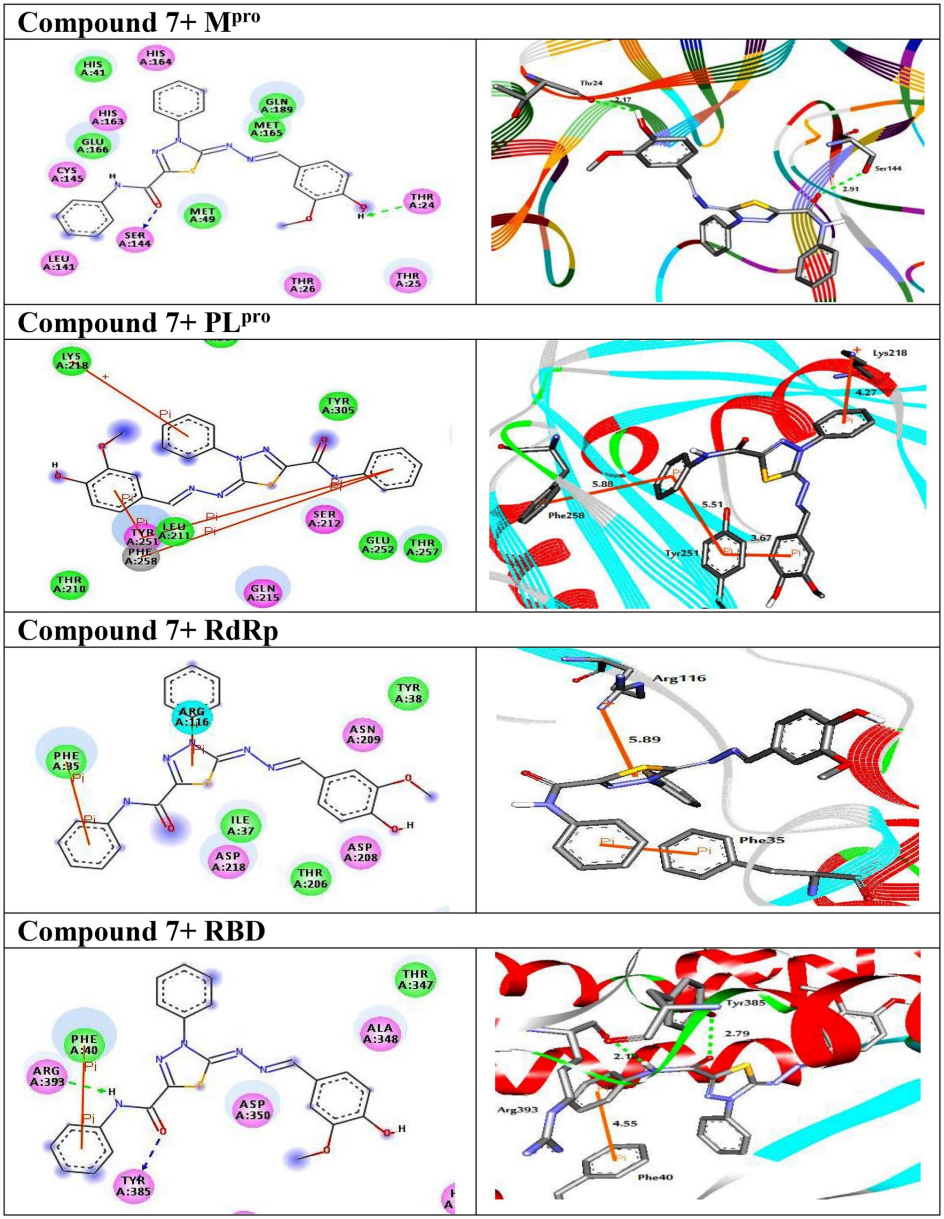
(Left side) 2D and (right side) 3D representations of intermolecular interactions of compound **7** against the active sites of SARS-CoV-2 (M^pro^), (PL.^pro^), (RdRp), and (RBD) of the spike protein. H-bond interactions are shown in green and blue dotted lines. π- interactions are shown in orange lines

In addition, most of the synthesized compounds demonstrated promising binding affinities against M^pro^ with binding energies ranged from −11.4 to −6.4 kcal/mol. The high docking scores of the studied compounds with M^pro^ would be returned to their ability to form hydrogen bonds, hydrophobic and van der Waals interactions with the amino acid residues of active sites (Fig. 5).

Compared to M^pro^, the examined compounds showed relatively weak binding affinities with PL^pro^, RdRp, and RBD, with docking scores ranged from −9.4 to −5.6, −8.2 to −5.2, and −6.8 to −5.3 kcal/mol, respectively. Interestingly, compound **7** displayed the highest binding affinity against all targets with docking scores of −11.4, −9.4, −8.2, and −6.8 kcal/mol, respectively. The promising binding affinity of **7** against the target M^pro^ is attributed to its ability to exhibit two hydrogen bond interactions with Thr24, and Ser144 at 2.17 and 2.91 Å, respectively (Fig. 4). Besides, inspecting the binding modes of compound **7** with PL^pro^, RdRp, and RBD unveiled its potentiality to form hydrogen bonds and Pi-stacking interactions, as presented in Fig. 4. The ligand molecule **7** docked with PL^pro^ amino acid residues (Lys218, Tyr251, and Phe258) through pi-stacked interactions at distances 4.27, 5.51, and 5.58 Å, respectively. Further, compound **7** interacted with the residues Arg116 and Phe35 of the target RdRp through pi-stacked interactions at distances 5.89, and 3.92 Å, respectively. Finally, it docked with RBD through two hydrogen bonds and one pi–pi interaction with the amino acid residues Tyr385, Arg393, and Phe40.

### Molecular dynamics (MD) simulations

Towards more reliable binding affinities, the molecular dynamics simulations were performed for all synthesized compounds in complex with SARS-CoV-2 targets. The binding energies (Δ*G*_binding_) were then calculated using the molecular mechanics-generalized born surface area (MM-GBSA) approach based on the collected snapshots for M^pro^, PL^pro^, RdRp, and RBD of spike protein over the production stage of 25 ns. The calculated MM-GBSA binding energies are listed in Table 2.

**Table 2:** Average MM-GBSA binding energies (in kcal/mol) over 25 ns for all synthesized compounds with the targets

**Compound**	**MM-GBSA Binding Energy (kcal/mol)**			
	**M**^**pro**^	**PL**^**pro**^	**RdRp**	**RDB**
2	−19.7	−25.9	−14.4	−5.1
3	−36.3	−22.9	−10.7	−15.7
4	−27.9	−27.9	−26.1	−21.7
5	−38.5	−26.6	−17.7	−13.9
6	−30.7	−22.8	−16.3	−13.1
7	−39.1	−15.8	−15.5	−14.9

It is apparent from Table 2 that the examined compounds with M^pro^ showed higher binding affinities over 25 ns MD simulations than with PL^pro^, RdRp, and RBD. The calculated MM-GBSA binding energies were in line with the predicted docking scores, demonstrating the high potency of the examined ligand molecules with M^pro^ over the other SARS-CoV-2 targets.

**Fig. 6:**
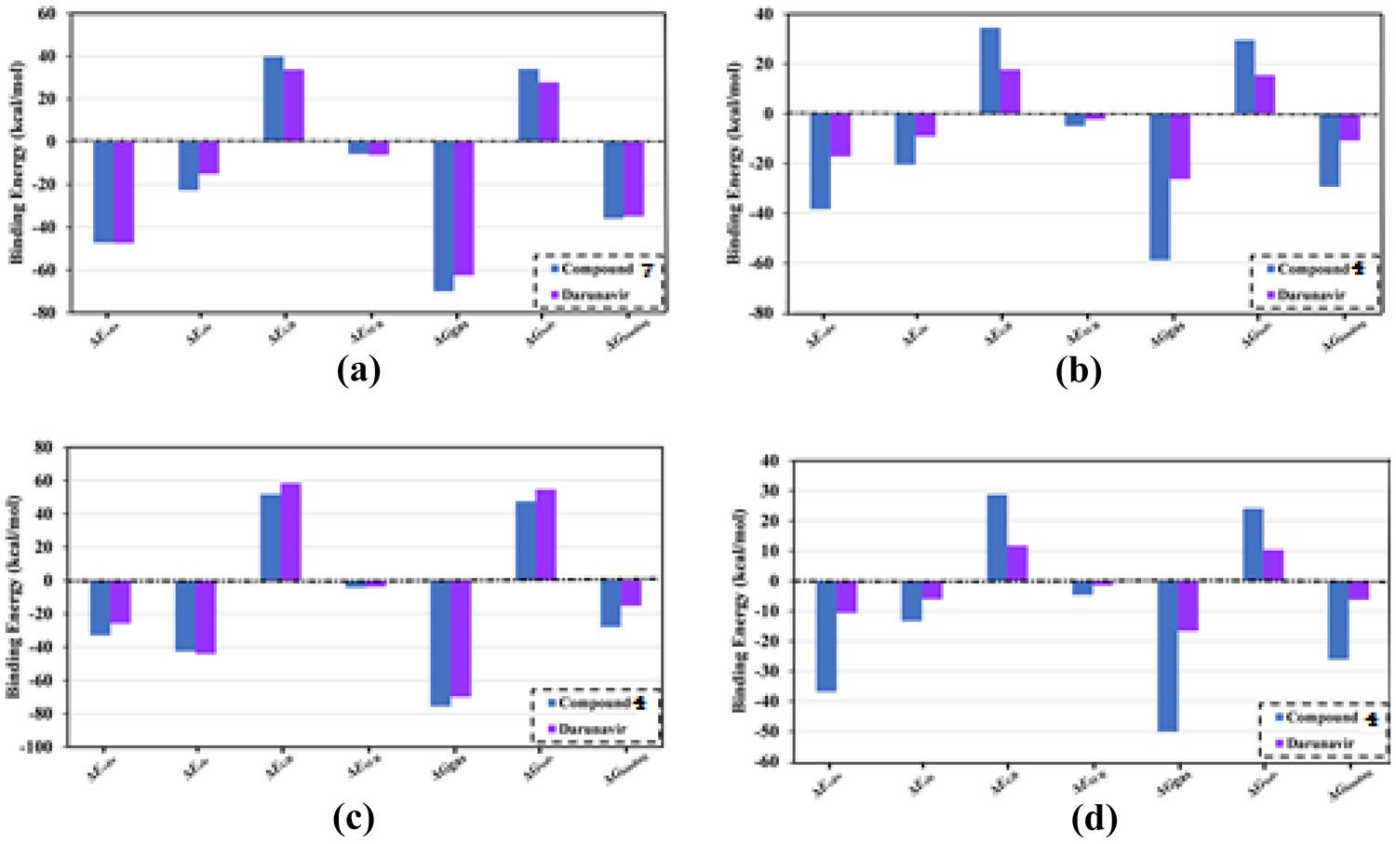
Decomposition of MM-GBSA binding energies for the investigated inhibitors in complex with of SARS-CoV-2 **a**) M^pro^, **b**) PL.^pro^, **c**) RdRp, and **d**) RBD of the spike protein throughout 100 ns MD simulations

Among the examined compounds, **7** exhibited the lowest binding energy with M^pro^ with a Δ*G*_binding_ value of −39.2 kcal/mol. Moreover, it showed weak binding energies of −15.8, −15.5, and −14.9 kcal/mol with PL^pro^, RdRp, and RBD, respectively. These results declared the selectivity of the compound **7** towards M^pro^ over PL^pro^, RdRp, and RBD. However, compound **4** demonstrated the lowest binding with PL^pro^, RdRp, and RBD with a Δ*G*_binding_ value of −27.9, −26.1, and −21.7 kcal/mol, respectively (Table 2). MD simulation for compound **7** in complex with M^pro^ and compound **4** complexed with PL^pro^, RdRp, and RBD were then elongated to 100 ns. Additionally, the corresponding MM-GBSA binding energy was calculated and was compared to the reference drug Darunavir (Fig. 6).

MM-GBSA binding energy of compound **7**-M^pro^, compound 4-PL^pro^, compound 4-RdRp, and compound 4-RBD complexes was decomposed to explore the predominant interactions between the investigated inhibitors and target. According to the data, it was found that the docking energy was calculated by *E*_vdw_ interactions with an average value of −47.2, −38.4, and −36.8 kcal/mol for investigated inhibitor with M^pro^, PL^pro^, and RdRp, respectively (Fig. 6). For compound **4** complexed with RdRp, the docking energy was dominated by *E*_ele_ interactions with an average value of −65.5 kcal/mol which was three times higher than that of Lopinavir and curcumin, with an average value of −42.5 kcal/mol (Fig. 6). Together these results demonstrated the promising binding affinity of compounds **7** and **4** with SARS-CoV-2 targets.

### Post-dynamics analysis

The interaction nature and stability of compound **7** and Darunavir within the active site of M^pro^ was estimated using structural and energetic analyses. Structural and energetic analyses including energy per**-**frame, centre-of-mass distance (CoM), root-mean-square deviation (RMSD), and root-mean-square fluctuation (RMSF) were performed over 100 ns MD simulations.

### Docking energy per frame

**Fig. 7:**
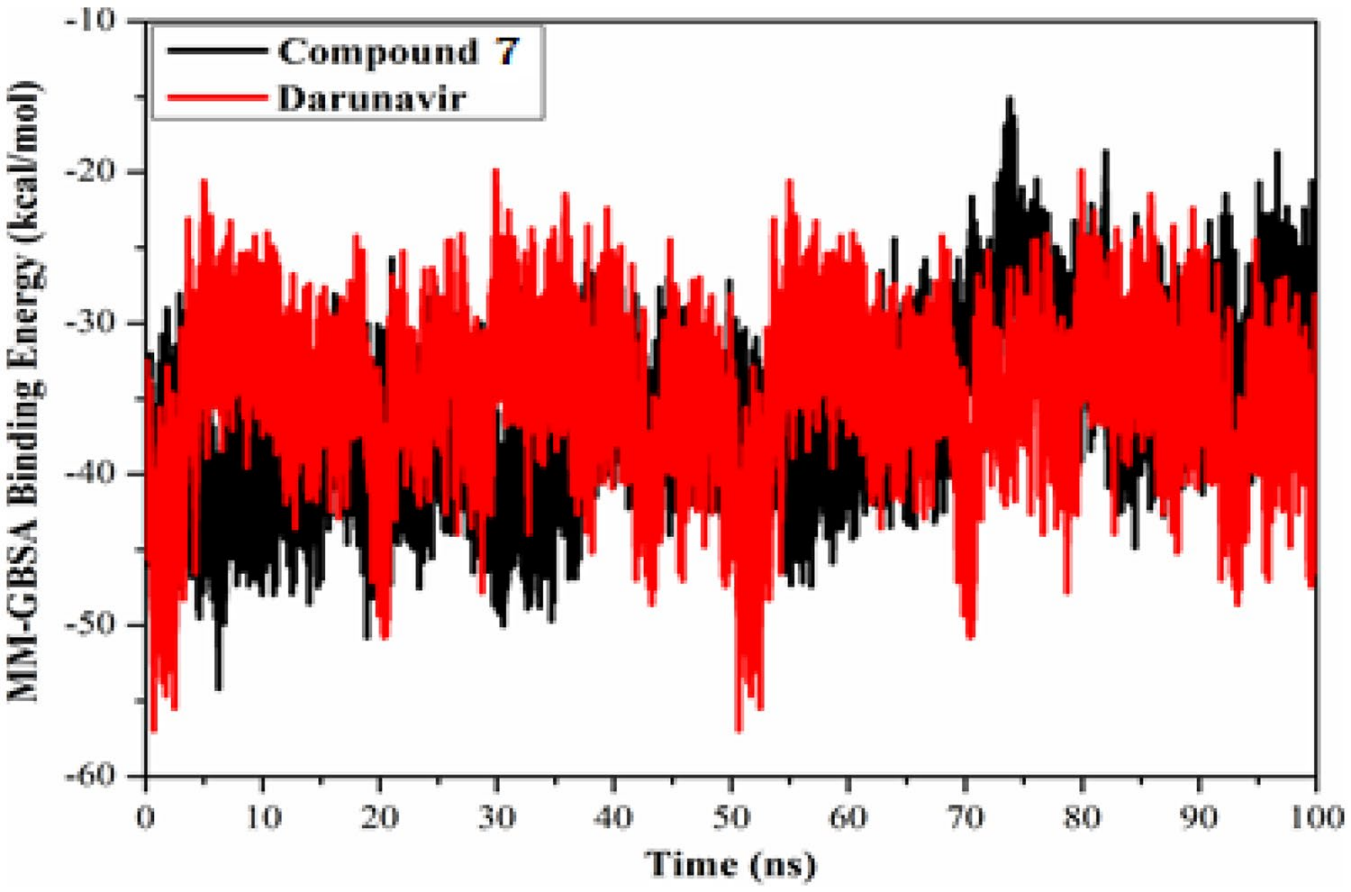
Variations in the MM-GBSA binding energies for compound **7** (in black), and Darunavir (in red) with SARS-CoV-2 M.^pro^ during 100 ns MD simulations

The stability of compound **7** and Darunavir in complex with SARS-CoV-2 M^pro^ was estimated using the correlation between the binding energy per-frame and time. MM-GBSA binding energy was subsequently evaluated per-frame for the most promising compound with each target and displayed in Fig. 7. The most interesting aspect of this graph is the overall stability of two identified compounds over 100 ns MD simulations with average values of −35.9, and −34.8 for compound **7**-M^pro^, and darunavir**-**M^pro^ complexes, respectively.

### Center-of-mass distance

**Fig. 8:**
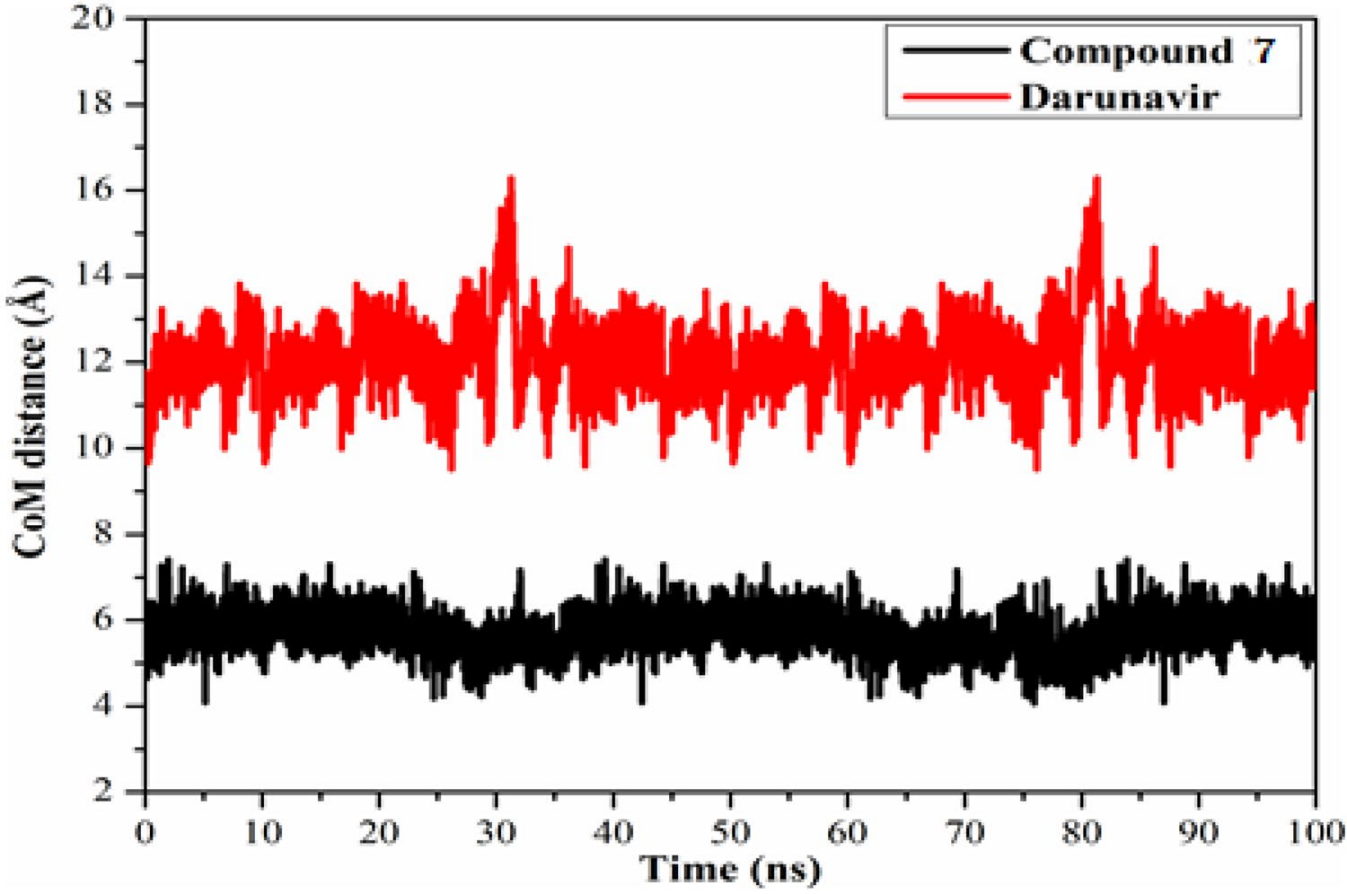
Centre-of-mass (CoM) distances between **7** (in black), and darunavir (in red) with GLU166 of SARS-CoV-2 M.^pro^ throughout 100 ns MD simulations

Interestingly, investigating the center-of-mass (CoM) distance between the compound **7**, and Darunavir and the residue Glu166 through the 100 ns MD simulations would reflect a strong indication of the high stability of the identified compounds inside the M^pro^ active site. The CoM distances were inspected over the 100 ns MD simulations and represented in Fig. 8. What stands out in Fig. 8 is the average CoM distance between the identified compounds and the key amino acid residue Glu166 was approximately constant, with average CoM distances of 5.7, and 12.1 Å, respectively. The current data revealed that compound **7** bound more tightly to the M^pro^ complex compared to Darunavir.

### Root-mean-square deviation

**Fig. 9:**
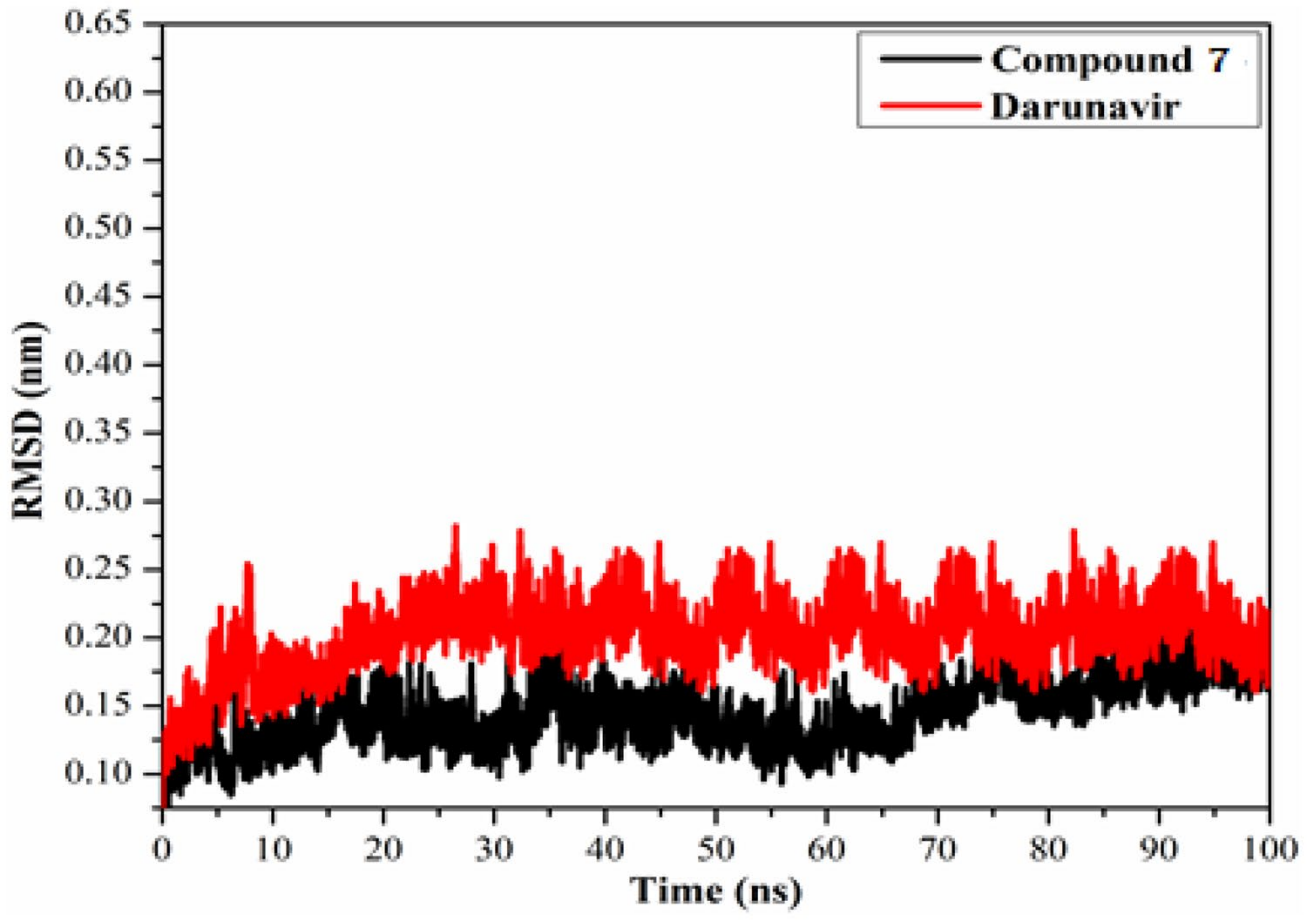
Root-mean-square-deviation (RMSD) of the backbone atoms from the initial structure for **7** (in black) and darunavir (in red) with the SARS-CoV-2 main protease (M.^pro^) over 100 ns MD simulations

The structural changes of **7**-M^pro^ and darunavir-M^pro^ complexes were evaluated using the root-mean-square deviation (RMSD). The conformational change of backbone atoms of the most promising three compounds in complex with SARS-CoV-2 M^pro^ has been compared with initial conformations over 100 ns MD simulations as shown in Fig. 9. As shown in Fig. 8, for **7-** M^pro^ and darunavir**-**M^pro^ complexes, the distance was noticed to be below 0.25 nm and the overall stability of these compounds inside the SARS-CoV-2 M^pro^ active site. These results confirmed that compound **7** is tightly bonded in the active site and does not affect the overall topology of SARS-CoV-2 M^pro^.

### ADMET and drug-likeness properties of the molecules

ADMET studies of the prepared molecules exhibited that they have acceptable absorption and distribution properties in the range of (91–80-98.76%) and (0.53–0.70), respectively. The physiochemical properties of the compounds exhibited acceptable values, as they have molecular weights and partition coefficients in the range of (256.35–445.49 g/mol), and < 5, respectively. Moreover, the molecules have no toxicity and carcinogenicity. All tested compounds showed good oral bioavailability within the body as they obeyed Lipinski's rule of five (Table 3).

**Table 3:** ADMET profile and drug-likeness properties of the docked molecules **2–7**

	**Molecular** **Weight** **(g/mol)**	**Blood–Brain** **Barrier** **(BBB +)**	**Caco-2 Permeability** **(Caco2 +)**	**%Human Intestinal** **Absorption (HIA +)**	**logp**	**TPSA** **A**^**2**^	**HBA**	**HBD**	**N** **rotatable**	**N** **violations**	**Volume** **A**^**3**^	**AMES toxicity**	**Carcinogenicity**
**Acceptable ranges**	**130–500**	**-3 to 1.2**	** < 25 poor** **500 great**	** > 80% high < 25% low**	** < 5**	** ≤ 140**	**2.0–20.0**	**0.0–6.0**	** ≤ 10**	** ≤ 1**	**500–2000**	**Nontoxic**	**Noncarcinogenic**
2	256.35	0.70	54.01	95.56	2.34	53.85	4	2	5	0	215.82	Nontoxic	Noncarcinogenic
3	398.44	0.64	55.39	92.45	3.50	98.32	7	1	7	0	340.20	Nontoxic	Noncarcinogenic
4	412.46	0.64	55.04	91.80	3.94	98.32	7	1	7	0	356.76	Nontoxic	Noncarcinogenic
5	368.41	0.63	52.03	98.76	3.19	89.09	6	1	5	0	314.41	Nontoxic	Noncarcinogenic
6	382.44	0.63	51.94	98.64	3.63	89.09	6	1	5	0	330.97	Nontoxic	Noncarcinogenic
7	445.49	0.53	52.53	98.50	4.18	101.11	6	2	7	0	381.66	Nontoxic	Noncarcinogenic

*HBA* number of hydrogen bond acceptors, *HBD* number of hydrogen bond donors, *logp* logarithm of partition coefficient between n-octanol and water, *n* *rotatable* number of rotatable bonds, *TPSA* topological polar surface area

## Conclusion

In this study, a new series of 1,3,4-thiadiazole derivatives was synthesized, characterized, and theoretically evaluated as Covid-19 inhibitors against four SARS-CoV-2 targets namely, main protease (M^pro^), papain-like protease (PL^pro^), RNA-dependent RNA polymerase (RdRp), and receptor-binding domain (RBD) of the spike protein. The molecular docking studies and molecular dynamics simulations exhibited the promising binding affinity of compound **7** with all targets. Therefore, it could be select as promising chemical moiety for designing of future inhibitors as anti-Covid-19 agents.

## Material and methods

### Instrumentation

All melting points were uncorrected and measured using electrothermal device. The IR spectra were recorded (KBr discs) using Shimadzu FT-IR 8201 PC spectrophotometer. ^1^H- and ^13^C-NMR spectra were recorded in (CD3)_2_SO solutions on a BRUKER 500 FT-NMR system spectrometer, and chemical shifts are expressed in ppm units using TMS as an internal reference. Mass spectra were recorded on a GC–MS QP1000 EX Shimadzu. Elemental analyses were carried out at the Microanalytical Center of Cairo University.

### Synthetic procedures of the target molecules (3–7)

A mixture of compound **2** (1.28 gm, 5 mmol) and the selected derivative of the hydrazonoyl halides (5 mmol) and 2–3 drops of DIPEA as a catalyst, were ground well in an open mortar with a pestle for 5–7 min. at RT till the mixture turned into melt. The grinding was continued for approximately 5–10 min, and the reaction was monitored by TLC. The solid was collected and washed with (water/ethanol) the recrystallized from the proper solvent to give the desired derivatives **3–7**, respectively.

#### “Ethyl 5-(4-hydroxy-3-methoxybenzylidene)hydrazono)-4-phenyl-4,5-dihydro-1,3,4-thiadiazole-2-carboxylate” (3)

Yellow crystals (95%); m.p. 172–174 °C, FT-IR (KBr, cm^−1^): *v* 1554 (C = C), 1599 (C = N), 1712 (C = O), 3471 (OH); ^1^H-NMR (DMSO-d_*6*_): *δ* 9.65 (s, 1H, OH), 8.29 (s, 1H, CH), 7.90 (d, 2H, *J* = 10 Hz, ArH), 7.33–7.45 (m, 4H, ArH), 7.17 (d, 1H, *J* = 10 Hz, ArH), 6.83 (d, 1H, *J* = 10 Hz, ArH), 4.15 (q, 2H, **CH**_**2**_CH_3_) 3.82 (s, 3H, OCH_3_),1.29 (t, 3H, CH_2_**CH**_**3**_);^13^C-NMR (100 MHz, DMSO-d_*6*_): *δ* 13.97 (CH_3_), 55.54 (O**CH**_**3**_), 62.75 (CH_2_), 110.18 (Ar.), 115.61(Ar.), 122.40 (Ar.), 122.56 (Ar.), 125.51 (Ar.), 127.32 (Ar.), 127.92 (Ar.), 129.07 (Ar.), 138.61 (Ar.), 142.22 (Ar.), 147.92 (Ar.), 149.51(CH), 156.12 (Ar.), 158.07 (Ar.), 163.75 (C = O); MS m/z (%): 398 (M^+^, 60). Anal. Calcd. for “C_19_H_18_N_4_O_4_S” (398): C, 57.28; H, 4.55; N, 14.06. Found: C, 57.34; H, 4.51; N, 14.01%.

#### “Ethyl 5-(4-hydroxy-3-methoxybenzylidene)hydrazono)-4-(*p*-tolyl)-4,5-dihydro-1,3,4-thiadiazole-2-carboxylate” (4)

Yellow crystals (92%); m.p. 180–182 oC, FT-IR: *v* 1550 (C = N), 1600 (C = N), 1705 (C = O), 3502 (OH); ^1^H-NMR: *δ* 9.65 (s, 1H, OH), 8.29 (s, 1H, CH), 7.76 (d, 2H, *J* = 10 Hz, Ar–H), 7.31–7.33 (m, 3H, Ar–H), 7.18 (d, 1H, *J* = 10 Hz, Ar–H), 6.84 (d, 1H, *J* = 10 Hz, Ar–H), 4.33 (q, 2H, CH_2_, **CH**_**2**_CH_3_), 3.81 (s, 3H, OCH_3_), 2.36 (s, 3H, CH_3_),1.30 (t, 3H, CH_2_**CH**_3_);^13^C-NMR: *δ* 13.97 (CH_3_), 20.64 (CH_3_), 55.53 (OCH_3_), 62.72 (CH_2_), 110.19 (Ar.), 115.58 (Ar.), 122.35 (Ar.), 122.56 (Ar.), 125.51 (Ar.), 129.46 (Ar.), 136.09 (Ar.), 136.91(Ar.), 141.88 (Ar.), 147.91 (Ar.), 149.55 (CH), 155.94 (Ar.), 158.08 (Ar.), 163.81 (C = O); MS m/z (%):412 (M^+^, 30). Anal. Calcd. for “C_20_H_20_N_4_O_4_S” (412): C, 58.24; H, 4.89; N, 13.58. Found: C, 58.19; H, 4.83; N, 13.53%.

#### “1-(5(-4-hydroxy-3-methoxybenzylidene)hydrazono)-4-phenyl-4,5-dihydro-1,3,4-thiadiazol-2-yl)ethan-1-one” (5)

Orange crystals (82%); mp. 212–214 oC, FT-IR: *v* 1550 (C = C), 1600 (C = N), 1678 (C = O), 3417 (OH); ^1^H-NMR: *δ* 9.65 (s, 1H, OH), 8.31 (s, 1H, CH), 7.34–7.97 (m, 6H, ArH), 7.16 (d, 1H, *J* = 10 Hz, ArH), 6.81(d, 1H, *J* = 10 Hz, ArH), 3.80 (s, 3H, OCH_3_), 2.48 (s, 3H, CH_3_); ^13^C-NMR: *δ* 25.14 (CH_3_), 55.54 (OCH_3_), 110.01 (Ar.), 115.59 (Ar.), 122.60 (Ar.), 125.49 (Ar.), 127.43 (Ar.), 129.13 (Ar.), 138.54 (Ar.), 147.87 (Ar.), 149.55 (CH), 150.17 (Ar.), 156.33 (Ar.), 164.04 (C = O); MS m/z (%): 368 (M^+^, 40). Anal. Calcd. for “C_18_H_16_N_4_O_3_S” (368): C, 58.68; H, 4.38; N, 15.21. Found: C, 58.63; H, 4.32; N, 15.16%.

#### “1-(5-(4-hydroxy-3-methoxybenzylidene)hydrazono)-4-(*p*-tolyl)-4,5-dihydro-1,3,4-thiadiazol-2-yl)ethan-1-one” (6)

Orange solid (81%); mp.191–193 °C, FT-IR: *v* 1541 (C = C), 1600 (C = N), 1681 (C = O), 3502 (OH); ^1^H-NMR: *δ* 9.64 (s, 1H, OH), 8.27 (s, 1H, CH), 7.80 (d, 2H, ArH), 7.30–7.32 (m, 3H, ArH), 7.13 (d, 1H, *J* = 10 Hz), 2.34 (s, 3H, CH_3_), 6.80 (d, 1H, *J* = 10 Hz, ArH), 3.79 (s, 3H, OCH_3_), 2.51 (s, 3H, CH_3_);^13^C-NMR: *δ* 20.66 (CH_3_), 24.95 (CH_3_), 55.52 (OCH_3_), 109.97(Ar.), 115.58 (Ar.), 122.53 (Ar.), 125.54 (Ar.), 129.48 (Ar.), 136.22(Ar.), 136.97 (Ar.), 147.94(Ar.), 149.53 (CH), 149.95 (Ar.), 156.07 (Ar.), 164.13 (C = O); MS m/z (%): 382 (M^+^, 15)%. Anal. Calcd. for “C_19_H_18_N_4_O_3_S” (382): C, 59.67; H, 4.74; N, 14.65. Found: C, 59.73; H, 4.70; N, 14.62%.

#### “5-(-4-hydroxy-3-methoxybenzylidene)hydrazono)-N,4-diphenyl-4,5-dihydro-1,3,4-thiadiazole-2-carboxamide” (7)

Yellow solid, m.p. 251–253 °C; yield (95%); FT-IR: *v* 1539 (C = C), 1600 (C = N), 1681 (C = O), 3337 (NH, OH);^1^H-NMR: *δ* 10.68 (s, 1H, NH), 9.65 (s,1H, OH), 8.36 (s, 1H, CH), 8.15 (d, 1H, J = 10 Hz, ArH), 7.75 (d, 1H, J = 10 Hz, ArH), 6.86–7.85 (m, 11H, ArH), 3.83 (s, 3H, OCH_3_);^13^C-NMR: *δ* 55.5 (OCH_3_), 110.05 (Ar.), 115.61(Ar.), 120.96 (Ar.), 122.34 (Ar.), 122.43 (Ar.), 124.70 (Ar.), 125.58 (Ar.), 126.94 (Ar.), 128.82 (Ar.), 128.93 (Ar.), 137.54 (Ar.), 138.94 (Ar.), 147.33 (Ar.), 147.95 (Ar.), 149.54 (CH), 155.89 (Ar.), 156.26 (Ar.), 164.16 (C = O); MS m/z [%]: 445 (M^+^), 444 (75), 317 (30), 281 (28), 255(80), 151(52), 127(48); Anal. Calcd. for “C_23_H_19_N_5_O_3_S” (445): C, 62.02; H, 4.30; N, 15.72%. Found: C, 62.06; H, 4.27; N, 15.65%.

### Computational methodology

#### Target identification

The crystal structures of SARS-CoV-2 main protease (M^pro^; PDB code: 6LU7) [19], papain-like protease (PL^pro^; PDB code: 6W9C) [20], RNA-dependent RNA polymerase (RdRp; PDB code: 6M71) [21], and receptor-binding domain (RBD) of spike protein (S-protein; PDB code: 6M0J) [22] were selected as templates for docking studies and molecular dynamics calculations. The water molecules, ions, and co-crystalized ligands if existing were removed [23]. Besides, the H + + server was utilized to investigate the protonation states of M^pro^, PL^pro^, RdRp, and RBD of S-protein, and all missing hydrogen atoms were added [24].

#### Inhibitor preparation

The chemical structures of the synthesized compounds were manually constructed, and their 3D structures were generated using Open Babel 2.4.1 tool [25–27]. All ligand molecules were then energetically minimized using the CHARMM Force Field [28].

#### Molecular docking

Molecular docking calculations [10, 12, 14, 18, 24, 29–33] were carried out using PyRx – virtual screening software [34]. The pdbqt files of M^pro^, PL^pro^, RdRp, and RBD of S-protein targets were prepared according to PyRx protocol. The docking algorithms were conserved to their default values, except the number of genetic algorithms (*GA*) run and the maximum number of energy evaluation (*eval*). In the current study, *GA* and *eval* were set to 250 and 25,000,000, respectively. The docking grid was set to 25 Å × 25 Å × 25 Å with a spacing value of 0.375 Å [17, 35]. The grid center was positioned at the center of the active site of M^pro^, PL^pro^, RdRp, and RBD of S-protein. The partial atomic charges of the examined compounds were estimated using the Gasteiger method [36]. The prediction of binding modes for each compound was handled using the built-in clustering analysis with an RMSD tolerance of 1.0 Å. Also, the lowest energy conformation from the largest cluster was picked out as a representative binding pose.

#### Molecular dynamics simulations

Molecular dynamics simulations were performed for the examined compounds in complex with the four studied SARS-CoV-2 targets using AMBER16 software. In MD simulations, the General AMBER force field (GAFF2) [37] and AMBER force field 14SB [38] were employed to describe the studied compounds and SARS-CoV-2 targets, respectively. The atomic partial charges of the examined compounds were calculated using the restrained electrostatic potential (RESP) approach at the HF/6 −31G* level with the help of Gaussian software [39]. Prior to RESP charge calculations, the studied compounds were first geometrically optimized at the B3LYP/6-31G* level of theory. The docked compound-target complexes were solvated in a cubic water box with 15 Å distances between the edges of the box and any atom of compound or compound-target complexes. The solvated compound-target systems were subsequently energy minimized for 5000 steps, gently annealed from 0 to 300 K over 50 ps, and equilibrated for 1 ns. The equilibrated systems were then simulated for 100 ns using periodic boundary conditions and NPT ensemble. The non-bonded cut-off distance was placed at 12 Å, and particle mesh Ewald (PME) method was applied to process long-range electrostatic interactions. The Langevin dynamics with the collision frequency gamma_ln set to 1.0 was used to conserve the temperature of the examined systems at 298 K. The pressure of the system was controlled using Berendsen barostat with a relaxation time of 2 ps. A time step of 2 fs and the SHAKE option to constrain all bonds involving hydrogen atoms were utilized. Coordinates and energy values were collected every 10 ps over the production stage for binding energy calculations and post-dynamics analyses. All MD simulations were conducted with the GPU of pmemd (pmemd.cuda) in AMBER16 on the CompChem GPU/CPU cluster (hpc.compchem.net). 2D and 3D visualization of the compound-targets interactions were performed using the Discovery studio software.

##### MM-GBSA binding energy

The binding free energies of the examined compounds with SARS-CoV-2 targets were estimated using molecular mechanical-generalized Born surface area (MM-GBSA) approach [40, 41]. For MM-GBSA calculations, uncorrelated snapshots were collected every 10 ps over the production stage. The MM-GBSA binding energy (${\Delta \mathrm{G}}_{\mathrm{binding}}$) can be conceptually summarized as:

$${\Delta \mathrm{G}}_{\mathrm{binding}}={\mathrm{G}}_{\mathrm{Complex }}-\left({\mathrm{G}}_{\mathrm{Compound}}+{\mathrm{G}}_{\mathrm{Enzyme}}\right)$$

where the energy term (*G*) is estimated as:

$$G={E}_{vdw}+{E}_{ele}+{G}_{GB}+{G}_{SA}$$

where *E*_vdw_ and *E*_ele_ are the van der Waals and electrostatic energies, respectively. *G*_GB_ is the electrostatic solvation free energy calculated from the generalized Born equation, and *G*_SA_ is the nonpolar contribution to the solvation free energy from the solvent-accessible surface area (SASA). Solute entropy contributions to binding energies were neglected, and a single-trajectory approach was employed, in which the coordinates of each molecule, receptor, and complex were isolated from a single trajectory.

#### ADMET analysis

The freely accessible online softwares such as admetSAR, SwissADME, and Mol inspiration are used to predict ADMET and drug-likeness properties of compounds.

## Supplementary Information

Below is the link to the electronic supplementary material.

Supplementary file1 (DOCX 2.23 MB)

## Data Availability

The data that support the findings of this study are included within the article and supplementary file.
