# Supplementary material for: In silico study to identify novel potential thiadiazole-based molecules as anti-Covid-19 candidates by hierarchical virtual screening and molecular dynamics simulations
Source: Struct Chem. 2022 Jun 15;33(5):1727–39. doi: 10.1007/s11224-022-01985-1 (PMC9198413; doi:10.1007/s11224-022-01985-1)
Supplement: Supplementary file 1 — Supplementary file1 (DOCX 2.23 MB) [file 11224_2022_1985_MOESM1_ESM.docx]

| **Compound 3**  **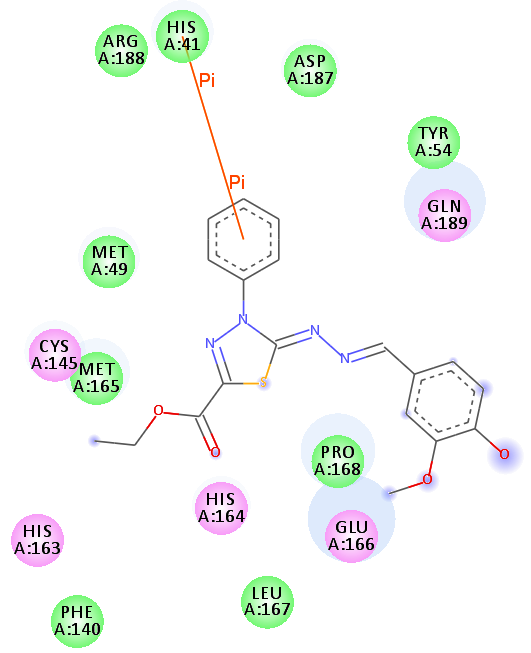** | **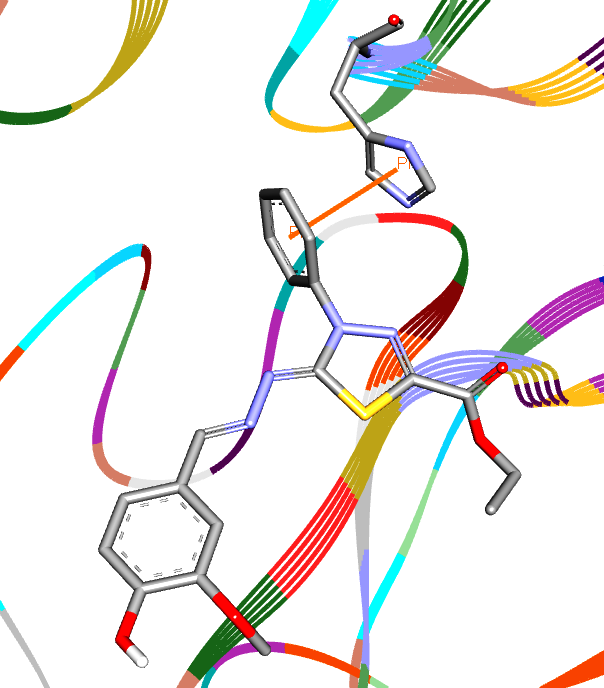** |
| --- | --- |
| **Compound 4**  **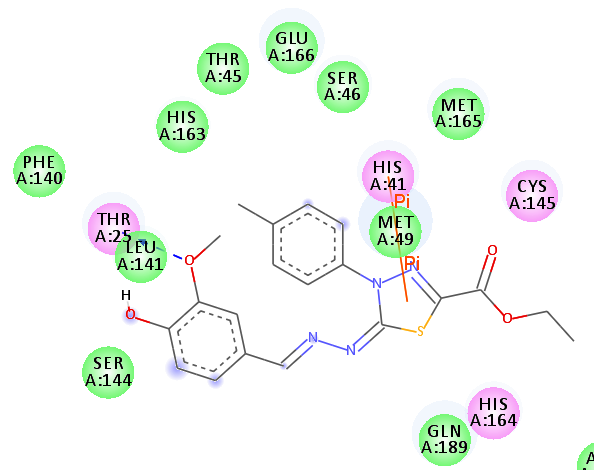** | **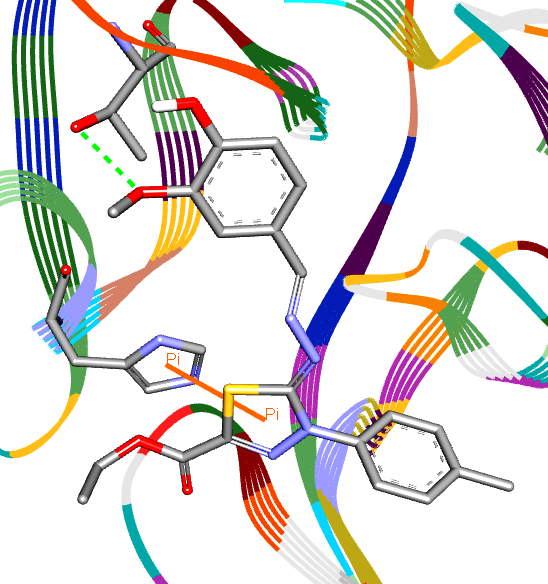** |
| **Compound 5**  **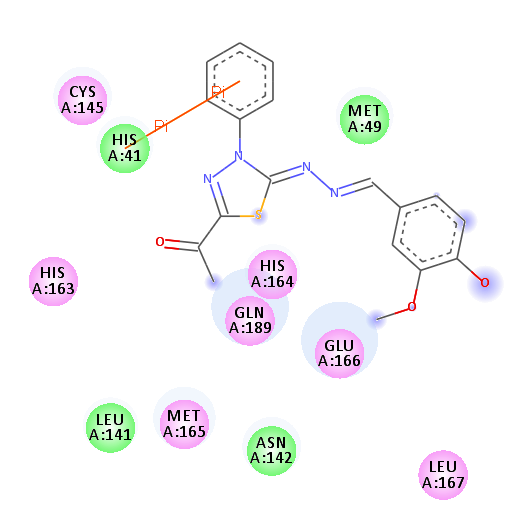** | 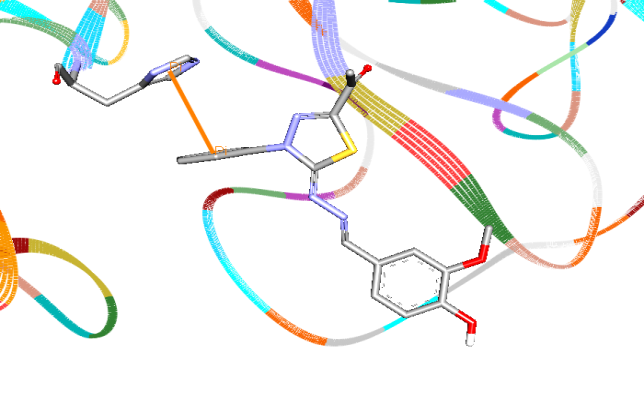 |
| **Compound 6**  **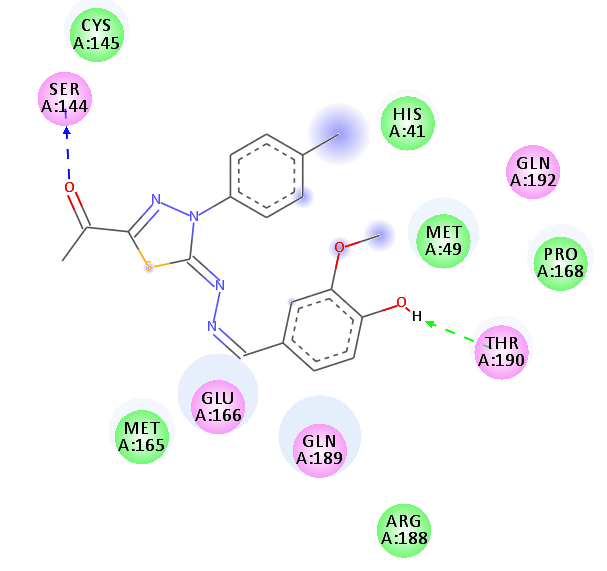** | **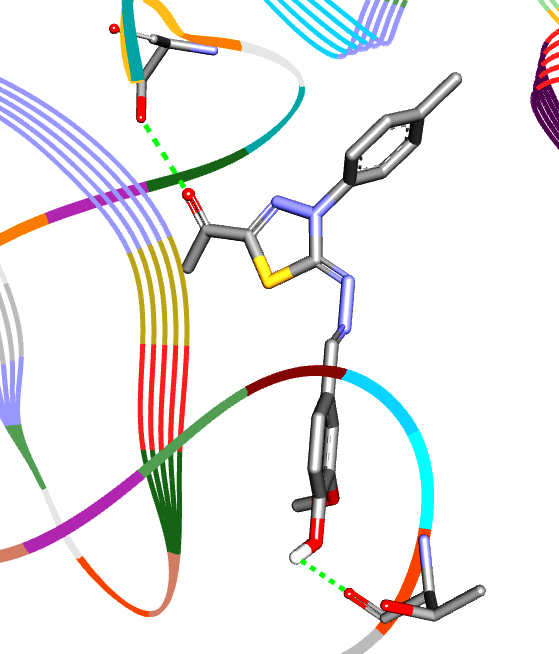** |

**Supplementary file**

**Figures S1.** (Left side) 2D and (right side) 3D representations of interactions of compounds 3-6 with amino acid residues of SARS-CoV-2 main protease (Mpro).

| **Compound 3**  **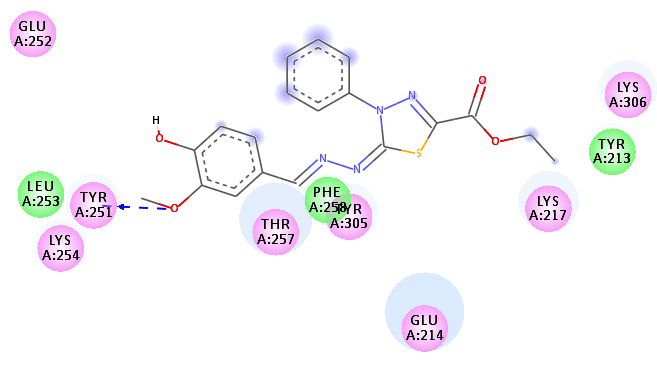** | **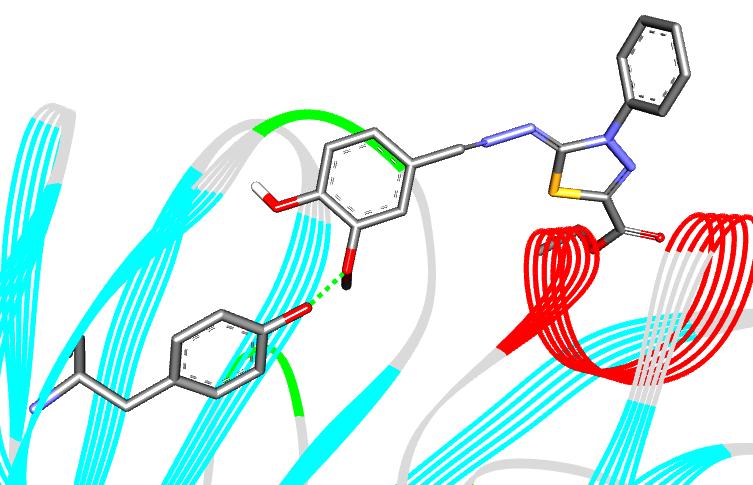** |
| --- | --- |
| **Compound 4**  **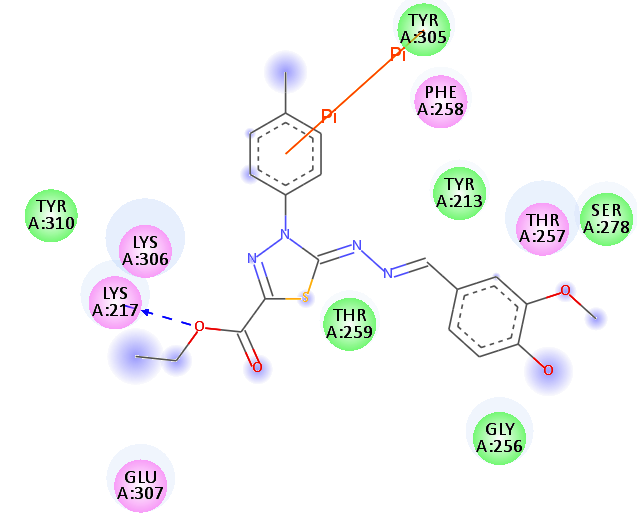** | **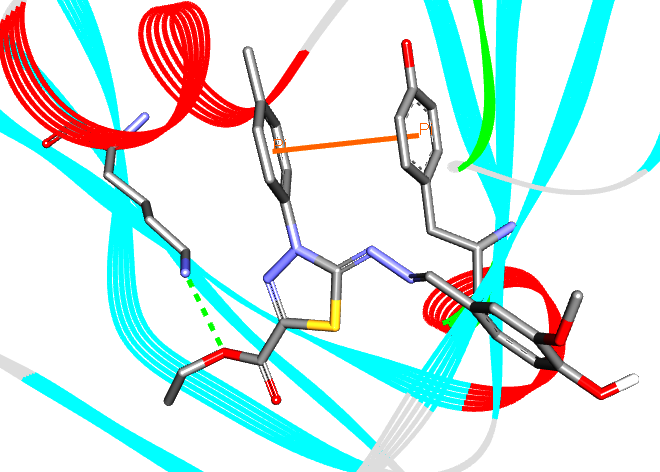** |
| **Compound 5**  **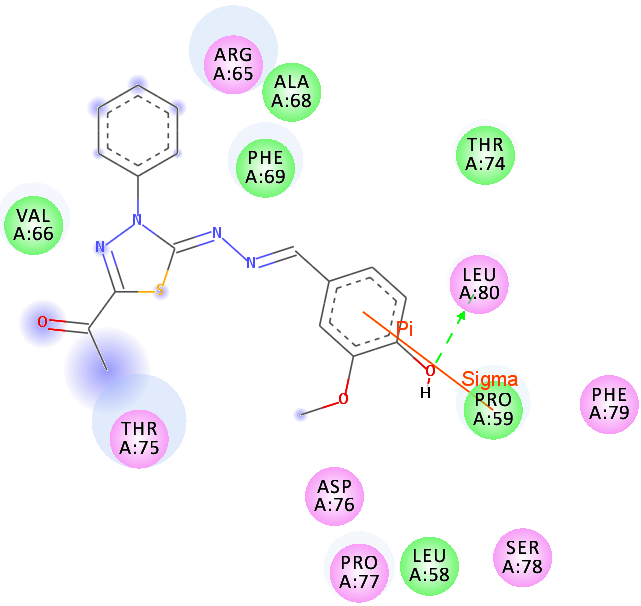** | **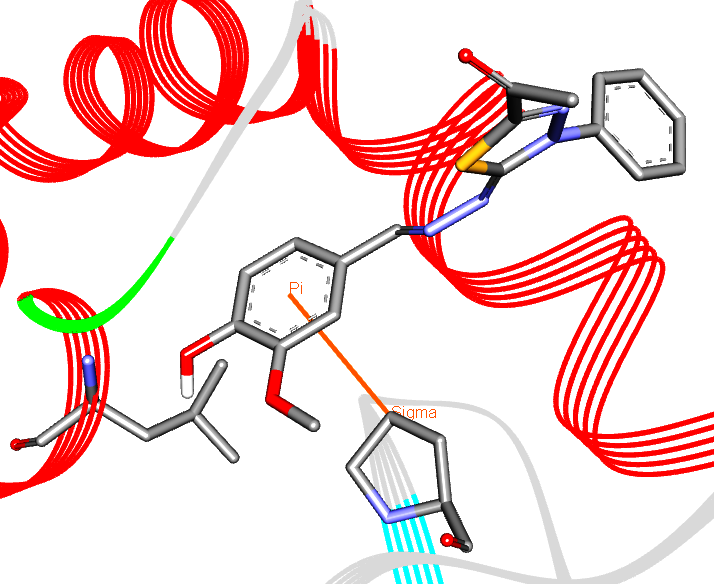** |
| **Compound 6**  **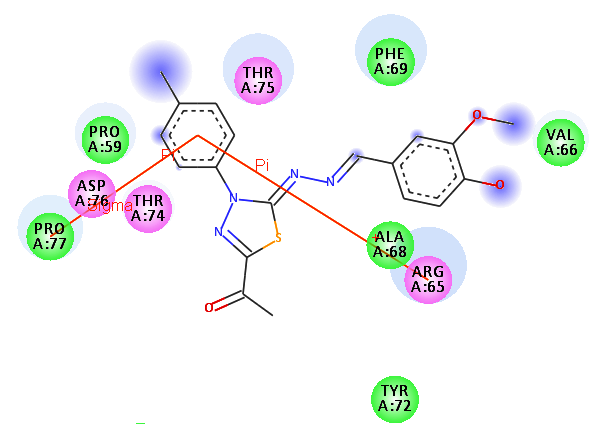** | **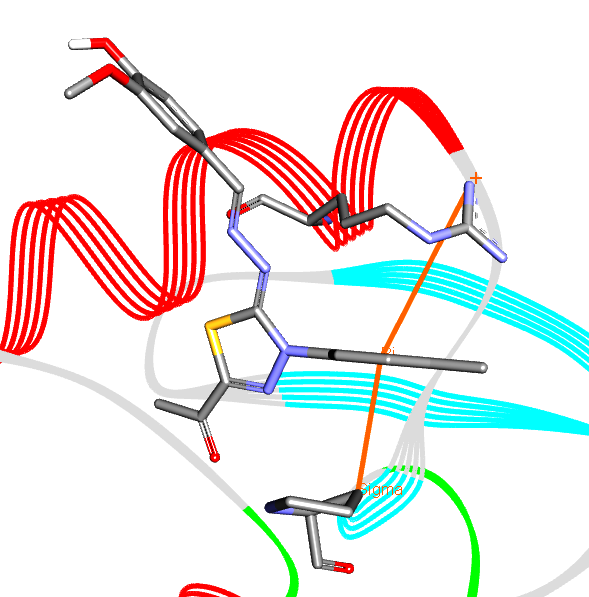** |

**Figures S2.** (Left side) 2D and (right side) 3D representations of interactions of compounds 3-6 with amino acid residues of SARS-CoV-2 papain-like protease (PL^pro^).

| **Compound 3**  **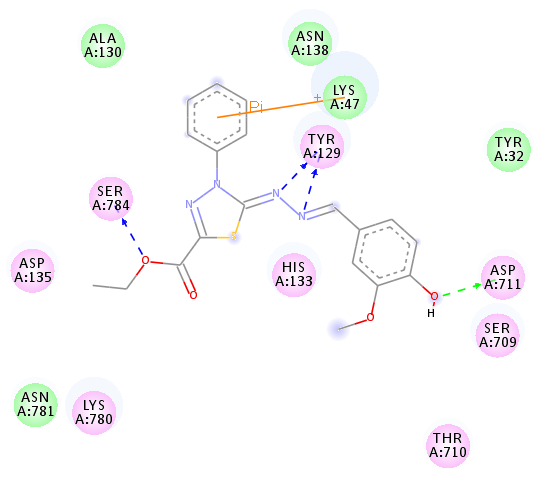** | **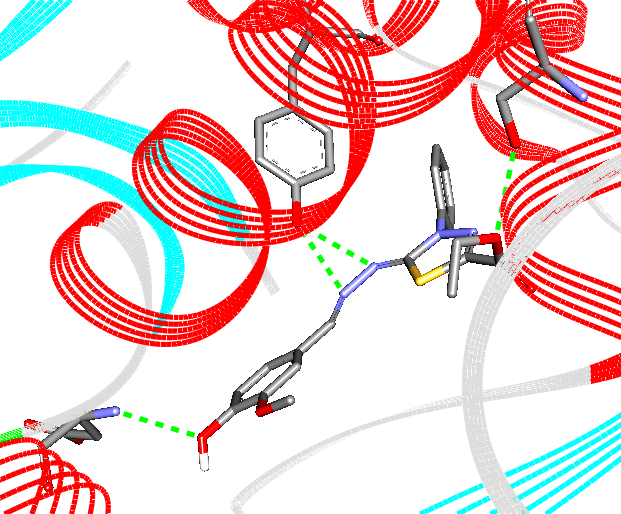** |
| --- | --- |
| **Compound 4**  **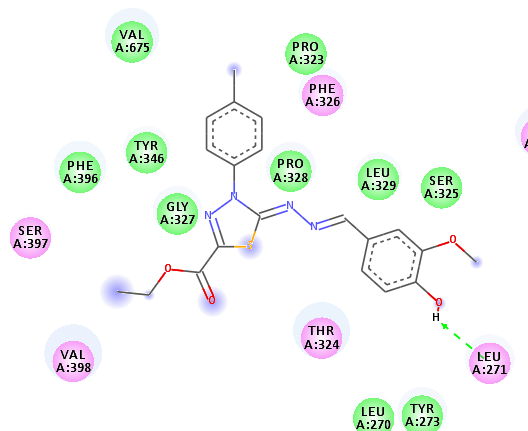** | **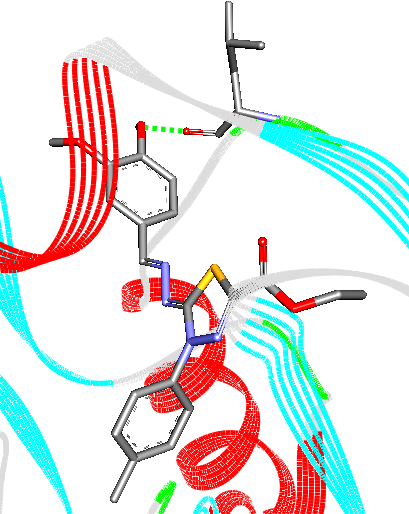** |
| **Compound 5**  **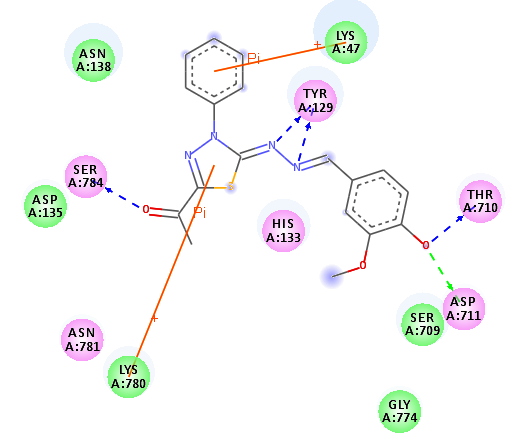** | **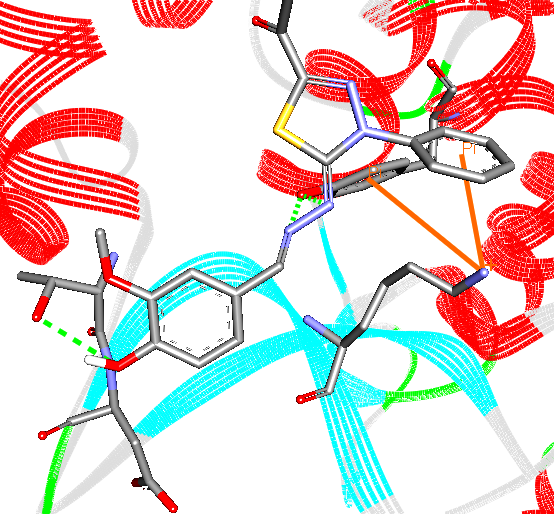** |
| **Compound 6**  **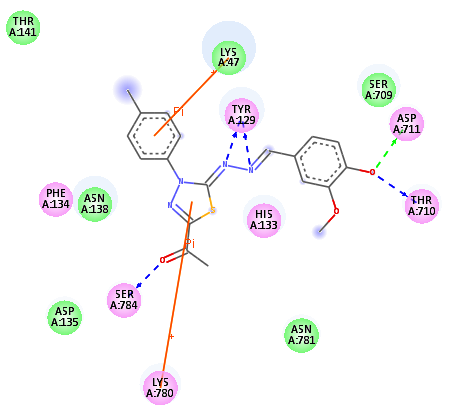** | **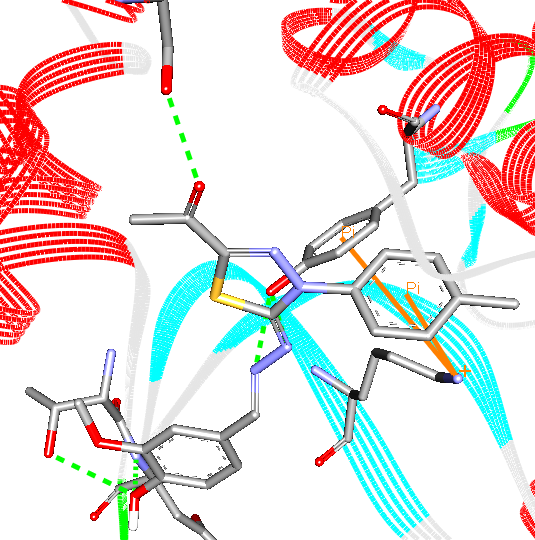** |

**Figures S3.** (Left side) 2D and (right side) 3D representations of interactions of compounds 3-6 with amino acid residues of SARS-CoV-2 RNA-dependent RNA polymerase (RdRp).

| **Compound 3**  **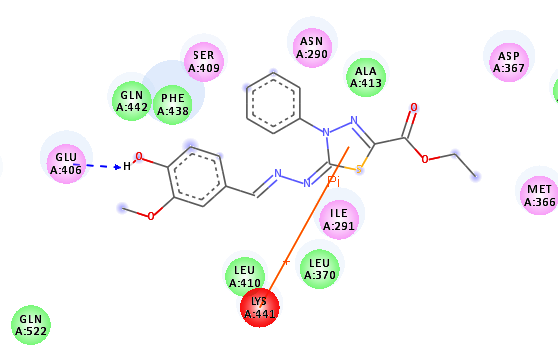** | **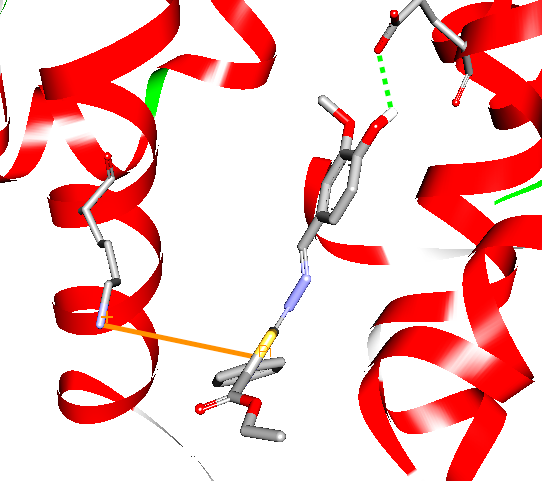** |
| --- | --- |
| **Compound 4**  **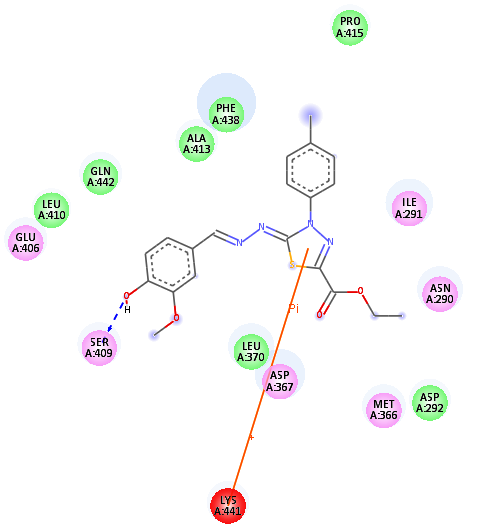** | **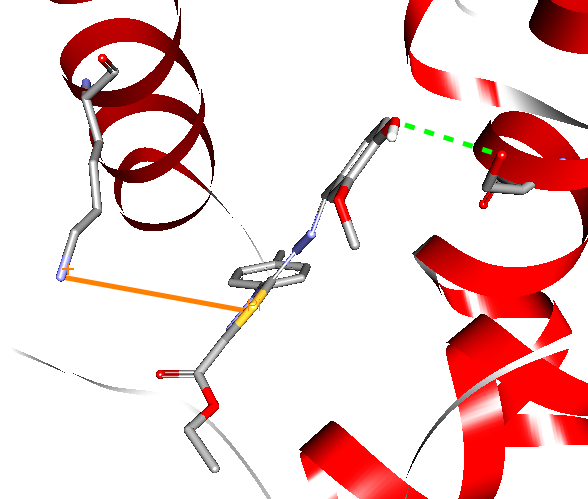** |
| **Compound 5**  **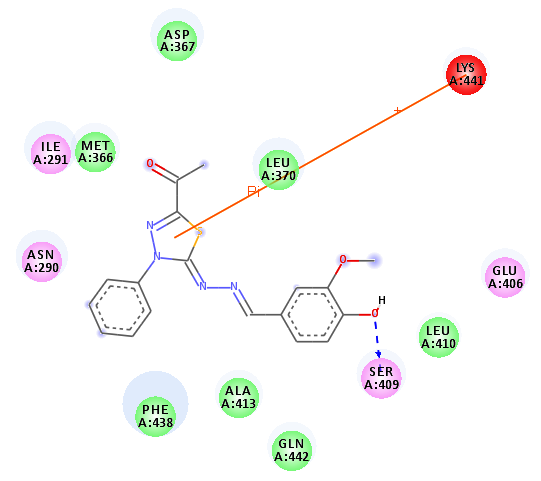** | **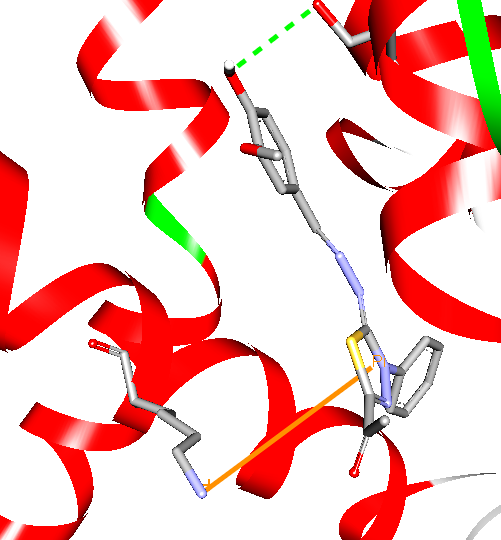** |
| **Compound 6**  **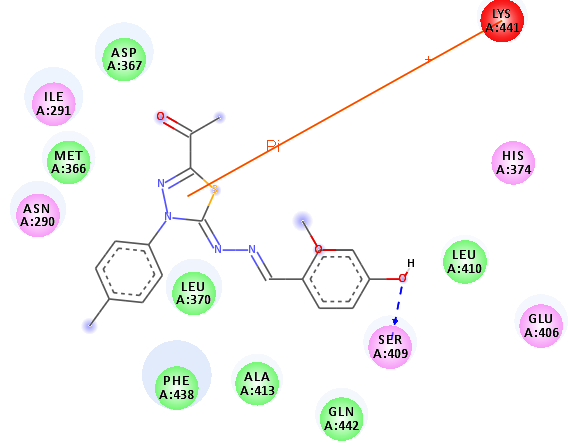** | **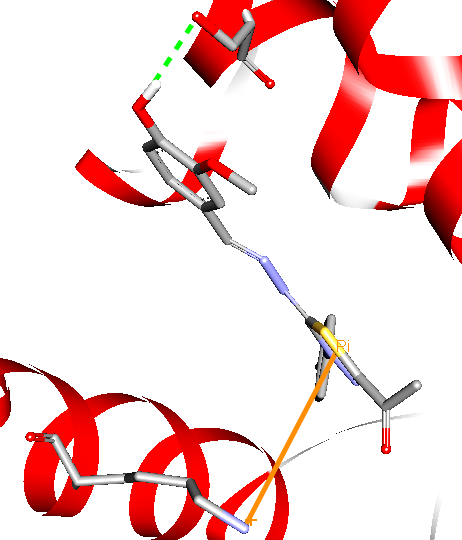** |

**Figures S4.** (Left side) 2D and (right side) 3D representations of interactions of compounds 3-6 with amino acid residues of SARS-CoV-2 receptor-binding domain (RBD) of the spike protein.
